# The effect of sugammadex sodium on muscle relaxation recovery in patients after suspension laryngoscopy surgery: A randomized controlled trial

**DOI:** 10.1097/MD.0000000000042385

**Published:** 2025-05-16

**Authors:** Qian Ge, Lin Shao, Chao Wen, Shiling Zhao

**Affiliations:** a Department of Anesthesiology, The Third People’s Hospital of Dalian, Dalian, Liaoning, China; b Department of Anesthesiology, The First Affiliated Hospital of Dalian Medical University, Dalian, Liaoning, China.

**Keywords:** fast-track anesthesia, remazolam, sugammadex sodium, suspension laryngoscope

## Abstract

**Background:**

Sugammadex sodium can antagonize aminosteroidal muscarinic drugs precisely and rapidly, so it has been widely used in fast-track anesthesia in recent years. However, it is not known whether there is an advantage of the antagonistic effect of sugammadex sodium over neostigmine at different doses and time points. In this single-center, randomized controlled study, we compared the effects of sugammadex sodium with neostigmine on postoperative myorelaxation recovery in patients undergoing suspension laryngoscopic surgery.

**Methods:**

A total of 90 patients scheduled for elective general anesthesia suspension laryngoscopy were selected, aged 18 to 65 years, body mass index 18 to 28 kg/m², and American Society of Anesthesiologists I–II grade. Patients were randomly divided into 3 groups: the sugammadex group (experimental groups, S_1_, S_2_), and the neostigmine group (control group, N), each comprising 30 patients. After the operation, group S_1_ received an intravenous injection of sugammadex sodium 2 mg/kg immediately, S_2_ received it when train-of-four COUNT (TOF-COUNT) > 2, and group N received intravenous injections of atropine 0.02 mg/kg + neostigmine 0.04 mg/kg when TOF-COUNT > 2. The mean arterial pressure, heart rate, pulse oxygen saturation, and bispectral index were recorded at various times: upon room entry (T_1_), during tracheal intubation (T_2_), at surgery start (T_3_), surgery end (T_4_), at extubation (T_5_), and upon room exit (T_6_). The duration of surgery, muscle relaxation recovery time from TOF-COUNT 0-2 at surgery end, and time from surgery end to extubation were recorded for each group, as well as the incidence of adverse reactions.

**Results:**

There were no statistically significant differences among the 3 groups in mean arterial pressure, heart rate, pulse oxygen saturation, and bispectral index at the 6 time points (T_1_, T_2_, T_3_, T_4_, T_5_, and T_6_). In terms of extubation timing, the S_1_ group showed a significantly shorter time compared with the S_2_ and N groups (*P* < .05). Compared with the S_2_ group, N group had significantly prolonged extubation times, showing a statistical difference. Compared with the N group, S_1_ and S_2_ groups had a significantly reduced incidence of bradycardia and increased secretions (*P* < .05).

**Conclusion:**

The use of sugammadex sodium in otolaryngological suspension laryngoscopy surgeries offers certain advantages over neostigmine in terms of muscle relaxation recovery. Administering sugammadex sodium 2 mg/kg directly after surgery as compared with waiting until TOF-COUNT > 2 allows for earlier removal of the tracheal tube without increasing adverse reactions.

## 1. Introduction

Fast-track anesthesia is an essential component of fast-track surgery. It is based on optimized anesthesia techniques and standardized anesthesia procedures, facilitating rapid recovery of vital organ functions from the anesthetic state, including during surgery.^[1]^ The advantages of fast-track anesthesia can improve the stability of the anesthesia process, shorten recovery times, reduce hospital stays, and decrease medical costs. Suspension laryngoscope surgery is characterized by short operation times and high stimulation intensity.^[2]^ It requires sufficient anesthesia depth, and patients often experience delays in extubation due to residual muscle relaxants. Traditional muscle relaxant antagonists, such as neostigmine, are less effective against deep muscle relaxation and often cause adverse reactions, making them unsuitable for fast-track procedures. Sugammadex sodium, a modified γ-cyclodextrin drug,^[3,4]^ forms a stable, inactive complex with aminosteroid muscle relaxants (like rocuronium or vecuronium), allowing for rapid and precise reversal of neuromuscular blockage, thereby achieving fast and accurate antagonism.

This study aimed to evaluate the different effects of sugammadex sodium and neostigmine at the end of suspension laryngoscope surgeries, comparing the impact of administering sugammadex sodium at different times on the removal of the tracheal tube. This research provides clinical evidence for fast-track anesthesia, accelerating patient recovery.

## 2. Materials and methods

### 2.1. General information

This study is a single-center, randomized controlled, single-blind trial. This study was reviewed and approved by the Ethics Committee of The Third People’s Hospital of Dalian, Liaoning Province (2023-091-001) and was registered in the Chinese Clinical Trial Registry (ChiCTR2400086028). Patients provided informed consent. A total of 90 patients scheduled for elective suspension laryngoscope surgery under general anesthesia at the Third People’s Hospital of Dalian, Liaoning Province were included. These patients were aged 18 to 65 years, body mass index of 18 to 28 kg/m^2^, and classified as American Society of Anesthesiologists I–II. Exclusion criteria included patients with significant preoperative respiratory or circulatory dysfunction, liver or kidney function abnormalities, mental disorders and communication barriers, allergies to rocuronium, benzodiazepines, or any component of sugammadex sodium, and those known or suspected to have neuromuscular diseases. Criteria for withdrawal or premature discontinuation of the study included voluntary withdrawal; surgery time > 20 minutes; and delayed recovery from anesthesia.

### 2.2. Grouping and treatment

Patients were randomly divided into 3 groups using a randomization table: sugammadex sodium group (experimental groups, S_1_, and S_2_), and neostigmine group (control group, N), each consisting of 30 patients. Routine electrocardiogram monitoring was conducted after entering the room, along with 3 L/min of oxygen inhalation via face mask. The mean arterial pressure (MAP), heart rate (HR), pulse oxygen saturation (SpO_2_), and bispectral index (BIS) were monitored. For anesthesia induction, midazolam 0.3 mg/kg and sufentanil 0.3 µg/kg were administered intravenously until the eyelash reflex disappeared. After calibrating the muscle relaxant monitor, rocuronium 0.6 mg/kg was injected intravenously, and tracheal intubation was performed when train-of-four COUNT (TOF-COUNT) ≤ 2. After confirming the tube placement, mechanical ventilation was started with tidal volume 6 to 8 mL/kg and EtCO_2_ 35 to 42 mm Hg. Anesthesia was maintained with continuous intravenous (IV) pumping of remazolam at 1.5 mg/kg/h (adjustable up to 3.0 mg/kg/h), keeping the BIS between 40 and 60. All surgeries were performed by the same surgeon and overseen by the same chief anesthesiologist. After the operation, group S_1_ received an IV injection of sugammadex sodium 2 mg/kg immediately, S_2_ received it when TOF-COUNT > 2, and group N received IV injections of atropine 0.02 mg/kg + neostigmine 0.04 mg/kg when TOF-COUNT > 2. All groups waited until the TOF ratio > 90% before stopping remazolam infusion and administering flumazenil 0.3 mg.

Perioperative management included IV injection of atropine 0.3 mg if HR < 50 bpm, and IV injection of ephedrine 3 mg if MAP ≤ 65 mm Hg. Nausea and/or vomiting were treated with IV drip of granisetron 3 mg.

### 2.3. Observation indicators

The MAP, HR, SpO_2_, and BIS were recorded for all groups upon room entry (T_1_), tracheal intubation (T_2_), start of surgery (T_3_), end of surgery (T_4_), extubation (T_5_), and room exit (T_6_). The duration of surgery, muscle relaxation recovery time from TOF-COUNT 0-2, and time from the end of surgery to extubation were recorded, along with the occurrence of adverse reactions (hypotension, allergic reactions, laryngospasm, bradycardia, nausea and/or vomiting, increased secretions).

### 2.4. Statistical analysis

Statistical analysis was performed using PASS15.0 software. On the basis of prior research,^[4]^ a significance level (*α*) of 0.05 and a power (1-β) of 0.90 were adopted, resulting in a required sample size of about 22 per group. Considering a dropout rate of approximately 20% (about 5 cases), the final sample size was set at 30 per group. Data were analyzed using SPSS 25.0 software. Normally distributed measurement data were expressed as mean ± standard deviation (*x* ± s), multiple group quantitative data were compared using the *F* test; rates among multiple groups were compared using the *χ*^2^ test or Fisher exact test; differences were considered statistically significant at *P* < .05.

## 3. Results

### 3.1. General information

This study initially included 90 patients, with 30 patients in each group. One patient in groups S_1_ and S_2_ were excluded because their surgery time exceeded 20 minutes. One patient in group N voluntarily withdrew from the study postoperatively and was therefore excluded. There were no statistically significant differences among the 3 groups in terms of gender, age, body mass index, and American Society of Anesthesiologists classification (*P* > .05), ensuring comparability among the groups (Table 1).

**Table 1:** General information comparison.

Group	Number	M/F	Age (years)	BMI (kg/m^2^)	ASA level I–II
S_1_	29	18/12	56.80 ± 10.78	24.44 ± 3.40	14/16
S_2_	29	17/11	56.90 ± 9.93	24.62 ± 4.14	12/16
N	29	16/13	56.90 ± 8.99	23.09 ± 4.49	15/14

ASA = American Society of Anesthesiologists, BMI = body mass index, F = female, M = male.

### 3.2. Hemodynamic indicators

For patients in groups S_1_, S_2_, and N, there were no statistically significant differences in MAP, HR SpO_2_, and BIS at the 6 time points T_1_, T_2_, T_3_, T_4_, T_5_, and T_6_ (*P* > .05), indicating that the hemodynamic response was stable across all groups throughout the various stages of the surgical and anesthesia procedures (Table 2).

**Table 2:** A comparison of the hemodynamic indicators at different time points for the 3 groups.

Feature	Group	T_1_	T_2_	T_3_	T_4_	T_5_	T_6_
MAP (mm Hg)	S_1_	99.59 ± 18.68	86.40 ± 11.94	80.31 ± 10.20	91.44 ± 7.91	98.76 ± 10.36	103.38 ± 6.48
	S_2_	95.59 ± 9.80	86.15 ± 9.85	79.78 ± 9.85	91.00 ± 8.12	98.29 ± 8.35	103.22 ± 5.77
	N	94.78 ± 11.49	86.50 ± 16.61	79.84 ± 10.04	91.36 ± 12.66	98.79 ± 12.31	103.35 ± 13.70
HR (bpm)	S_1_	102.91 ± 8.72	95.21 ± 5.11	85.62 ± 4.41	89.62 ± 4.42	87.41 ± 4.40	87.62 ± 4.53
	S_2_	104.01 ± 8.02	92.92 ± 8.93	84.51 ± 8.82	88.51 ± 8.84	86.32 ± 8.81	86.51 ± 8.99
	N	99.92 ± 4.63	89.71 ± 3.73	83.51 ± 7.31	87.53 ± 7.31	85.21±7.32	85.52 ± 7.66
SpO_2_ (%)	S_1_	97.21 ± 2.22	96.81 ± 1.70	97.11 ± 1.51	98.55 ± 1.57	99.01 ± 0.81	98.15 ± 1.81
	S_2_	98.14 ± 1.62	97.81 ± 1.54	98.92 ± 0.52	97.64 ± 1.54	99.21 ± 0.82	97.25 ± 1.71
	N	98.51 ± 1.49	98.02 ± 1.31	98.32 ± 1.68	98.21 ± 1.41	99.01 ± 0.79	97.66 ± 2.01
BIS	S_1_	95.81 ± 1.91	48.32 ± 2.01	56.92 ± 2.02	56.07 ± 2.84	75.42 ± 2.31	93.02 ± 4.22
	S_2_	96.23 ± 2.33	45.56 ± 3.03	56.83 ± 1.78	56.12 ± 2.35	75.02 ± 3.59	92.81 ± 4.01
	N	96.02 ± 2.01	48.23 ± 2.06	57.46 ± 1.70	55.08 ± 2.02	76.02 ± 4.81	93.17 ± 3.62

BIS = bispectral index, HR = heart rate, MAP = mean arterial pressure, SpO_2_ = pulse oxygen saturation.

### 3.3. Surgery duration, TOF-COUNT 0-2 muscle relaxation recovery time and extubation time

There was no statistical difference in surgery duration among patients in groups S_1_, S_2_, and N, indicating uniformity in the length of the surgical procedures across the groups. There were also no significant differences between groups S_2_ and N in terms of TOF-COUNT 0-2 muscle relaxation recovery time (*P* > .05), suggesting similar effectiveness of the neuromuscular blockade reversal for these 2 groups when using their respective antagonists. However, in terms of extubation time, there was a statistically significant difference. The extubation time for group S_1_ was significantly shorter compared with groups S_2_ and N (*P* < .05). In addition when comparing group N to group S_2_, the extubation time was significantly longer for group N (*P* < .05). These differences highlight the efficacy of sugammadex sodium in group S_1_ in enabling faster recovery and extubation compared with neostigmine and the delayed administration in group S_2_ (Table 3).

**Table 3:** The comparative data for surgery time, TOF-COUNT 0-2 muscle relaxation recovery time, and extubation time among the 3 groups.

Group	Surgery time (min)	TOF-COUNT 0-2 muscle relaxation recovery time (min)	Extubation time (min)
S_1_	6.70 ± 1.70	–	4.30 ± 0.95
S_2_	6.40 ± 2.00	18.70 ± 2.11	23.20 ± 2.50*
N	6.40 ± 1.84	19.00 ± 3.13	23.80 ± 4.69*^,^†

TOF-COUNT = train-of-four COUNT.

* Compared with S_1_ group, *P* < .05.

† Compared with S_2_ group, *P* < .05.

### 3.4. Perioperative adverse events

Compared with group N, the incidence of bradycardia and increased secretions was significantly lower in groups S_1_ and S_2_ (*P* = .012, *P* = .000, respectively), suggesting that the use of sugammadex sodium in groups S_1_ and S_2_ might be associated with a better safety profile regarding these specific complications, compared with the use of neostigmine in group N. This outcome supports the potential benefits of sugammadex sodium in reducing certain perioperative adverse events, aligning with its pharmacological profile which is known for fewer cardiovascular side effects compared with traditional antagonists like neostigmine (Table 4).

**Table 4:** Detailed information on the occurrence rates of these adverse events among the 3 groups.

Group	Number	Hypotension	Allergic reactions	Laryngospasm	Bradycardia	Nausea/vomiting	Increased secretions
S_1_	29	0 (0)	0 (0)	0 (0)	0 (0)	1 (3)	1 (3)
S_2_	29	0 (0)	0 (0)	0 (0)	1 (3)	0 (0)	2 (6)
N	29	0 (0)	0 (0)	1 (3)	6 (20)	0 (0)	9 (30)

Bradycardia *P* = .012 and hypersecretion *P* = .000.

## 4. Discussion

In recent years, due to changes in lifestyle habits and social–environmental factors, the incidence of benign lesions in the glottic area has been steadily increasing. Commonly observed benign vocal cord lesions include vocal cord polyps, epiglottic cysts, and vocal nodules,^[5]^ though their pathogenesis is not yet fully understood. Suspension laryngoscopy is one of the primary surgical interventions for treating benign lesions in the glottic area. This surgery is characterized by its short duration, highly stimulating maneuvers, and high requirements for muscle relaxation. Aminosteroid neuromuscular blockers, such as rocuronium, are favored by anesthesiologists due to their strong relaxation effects on pharyngeal muscles.^[6,7]^ Although aiming for rapid onset of muscle relaxants, quick recovery postsurgery presents new challenges. Currently, clinical practices such as predosing techniques and the combined use of inhalational anesthetics are employed to reduce the dosage of muscle relaxants during surgery and accelerate postoperative recovery. However, many patients still experience residual effects of muscle relaxants after surgery, necessitating the use of muscle relaxant antagonists for reversal. Traditional drugs like neostigmine can cause perioperative adverse events such as sinus bradycardia, increased secretions, nausea, and vomiting. Thus, there is a need to seek new perioperative anesthesia management strategies to optimize the management of muscle relaxants after general anesthesia for suspension laryngoscopy. This approach aims to provide a theoretical basis for fast-track anesthesia in otolaryngology and accelerate patient recovery.

Sugammadex sodium, a chemically modified γ-cyclodextrin, forms a stable 1:1 complex with free neuromuscular blocking agents (such as rocuronium or vecuronium) in the plasma.^[8]^ This rapidly reduces the level of free neuromuscular blockers in the plasma, creating a concentration gradient that shifts the neuromuscular blockers from the neuromuscular junction back into the plasma. The free neuromuscular blockers in the plasma are then encapsulated by free sugammadex, allowing for precise antagonism after surgery. This not only shortens the time to extubation but also reduces the occurrence of perioperative complications, aligning with the concept of rapid recovery.^[9]^ Studies by researchers such as Moss et al^[10]^ have shown that the use of sugammadex sodium can effectively shorten the time patients spend in the operating and recovery rooms, thus speeding up patient turnover. Research by Gu et al^[11]^ and others have confirmed that sugammadex sodium can effectively shorten the time to extubation compared with neostigmine, without being limited by the type of surgery, supporting the findings of this study. In this study, both the S_1_ and S_2_ groups used sugammadex sodium for muscle relaxant antagonism after surgery, resulting in significantly shorter extubation times compared with the N group. The S_1_ group, which received 2 mg/kg of sugammadex sodium immediately after surgery, showed a statistically significant difference in extubation time compared with the S_2_ group, which waited until the TOF-COUNT reached 2 before administering the antagonist. This suggests that immediate antagonism postsurgery is more effective than waiting for partial recovery of muscle function. The dose of sugammadex sodium used in this study was 2 mg/kg, which is less than the dosage recommended in the product literature. The results confirm that this dose not only adequately reversed the muscle relaxants but also reduced medical costs during the patient’s hospitalization.

During the perioperative period, traditional muscle relaxant antagonists like neostigmine, an anticholinesterase drug, can affect acetylcholinesterase at the neuromuscular junction and other sites. This can cause bradycardia, increased gland secretion, and visceral smooth muscle spasms.^[12,13]^ When using neostigmine, it is often necessary to coadminister anticholinergic drugs, such as atropine or glycopyrrolate, to counteract the muscarinic adverse effects on the cardiovascular system, airways, and intestines. Due to the differences in onset times of these drugs, there can be a clinical phenomenon of an initial increase and subsequent decrease in HR, increasing the incidence of cardiovascular events. On the other hand, the use of sugammadex sodium rarely results in sinus bradycardia,^[14,15]^ consistent with the conclusions of this study. Research by Huo et al^[16]^ suggests that sugammadex sodium may trigger allergic reactions. However, other studies report that perioperative allergic reactions are not definitively linked to the use of sugammadex sodium.^[17]^ In this study, none of the 87 participants experienced allergic reactions related to the antagonist during the perioperative period, which could be attributed to the lower doses used or the small sample size, necessitating further observation.^[18]^ Regarding perioperative nausea and vomiting (PONV), studies by Chang et al^[19]^ and others have found that the incidence of nausea and vomiting is significantly reduced after reversing muscle relaxants with sugammadex sodium compared with neostigmine. Similar findings suggest that sugammadex reduces the rate of PONV in otolaryngological surgeries compared with neostigmine.^[20]^ However, some researchers have found that the use of sugammadex sodium does not improve the incidence of PONV postoperatively.^[21,22]^ In this study, the occurrence of nausea and vomiting postoperatively among the 3 groups showed no statistical significance, indicating that further experimental research is needed to confirm these findings and potentially refine the management of PONV in clinical settings.

In summary, sugammadex sodium has shown greater advantages over neostigmine in the recovery from muscle relaxation following suspension laryngoscope surgeries. It significantly shortens the time to extubation and reduces the incidence of bradycardia. It is recommended to administer sugammadex sodium via IV injection immediately after surgery for optimal effects. However, this study is a single-center experiment and has limitations such as small sample size and insufficient differentiation in sugammadex dosage groups. Further research is needed to refine these findings and provide more comprehensive guidance for clinical practice.

## Author contributions

**Data curation:** Qian Ge, Lin Shao.

**Formal analysis:** Qian Ge.

**Investigation:** Lin Shao.

**Supervision:** Chao Wen, Shiling Zhao.

**Software:** Chao Wen.

**Project administration:** Shiling Zhao.

**Writing – original draft:** Qian Ge.
